# Long-Term Effect of Docosahexaenoic Acid Feeding on Lipid Composition and Brain Fatty Acid-Binding Protein Expression in Rats

**DOI:** 10.3390/nu7105433

**Published:** 2015-10-22

**Authors:** Marwa E. Elsherbiny, Susan Goruk, Elizabeth A. Monckton, Caroline Richard, Miranda Brun, Marwan Emara, Catherine J. Field, Roseline Godbout

**Affiliations:** 1 Department of Oncology, Cross Cancer Institute, University of Alberta, 11560 University Avenue, Edmonton, AB T6G 1Z2, Canada; marwae@ualberta.ca (M.E.E.); eamonckton@shaw.ca (E.A.M.); mbrun@ualberta.ca (M.B.); 2 Department of Agricultural, Food and Nutritional Science, University of Alberta, Edmonton, AB T6G 2E1, Canada; sgoruk@ualberta.ca (S.G.); cr5@ualberta.ca (C.R.); cjfield@ualberta.ca (C.J.F.); 3 Centre for Aging and Associated Diseases, Zewail City for Science and Technology, Cairo 12588, Egypt; memara@zewailcity.edu.eg

**Keywords:** arachidonic acid, brain development, brain lipids, diet and dietary lipids, fatty acid/binding protein

## Abstract

Arachidonic (AA) and docosahexaenoic acid (DHA) brain accretion is essential for brain development. The impact of DHA-rich maternal diets on offspring brain fatty acid composition has previously been studied up to the weanling stage; however, there has been no follow-up at later stages. Here, we examine the impact of DHA-rich maternal and weaning diets on brain fatty acid composition at weaning and three weeks post-weaning. We report that DHA supplementation during lactation maintains high DHA levels in the brains of pups even when they are fed a DHA-deficient diet for three weeks after weaning. We show that boosting dietary DHA levels for three weeks after weaning compensates for a maternal DHA-deficient diet during lactation. Finally, our data indicate that brain fatty acid binding protein (FABP7), a marker of neural stem cells, is down-regulated in the brains of six-week pups with a high DHA:AA ratio. We propose that elevated levels of DHA in developing brain accelerate brain maturation relative to DHA-deficient brains.

## 1. Introduction

Human brain development starts at the fifth postmenstrual week and continues after birth with most of the brain’s neurobiological processes fully developed by adolescence [1,2]. There is a spurt in brain growth during the last trimester of pregnancy, with the mass of the brain approaching that of adult brain by the time the child is about three years old [3]. The rapid growth of the brain during the last trimester requires a significant supply of long chain polyunsaturated fatty acids (PUFA), especially docosahexaenoic acid (DHA, C22:6, ω-3) and arachidonic acid (AA, C20:4, ω-6) which are the major PUFA components of brain lipids [4].

DHA accumulates in brain gradually over the course of its development specifically from the third trimester onwards [5]. Analysis of the phosphatidylethanolamine component of brain phospholipids revealed increases in ω-3 PUFA, contributed mainly by DHA, and in the ω-3:ω-6 PUFA ratio, in brains of children from six months to 8 years old compared to brains of zero to six month old infants. In turn, the latter had a higher ω-3 PUFA content and ω-3:ω-6 PUFA ratio than fetuses at 26 to 42 weeks of gestation [6]. Similarly, when rat brains were examined at postnatal day (P) 8 (comparable to 36–40 weeks of gestation in humans in terms of brain maturation [7]) and at embryonic days (E) 17 and E20, an increase in ω-3 PUFA was observed, with most of the increase being accounted for by DHA [8]. A similar scenario was reported for developing piglets, with increased DHA content at term and 14 weeks postnatally compared to mid-gestation [9].

Long chain ω-3 and ω-6 PUFAs can be endogenously synthesized from their precursors, alpha-linolenic acid (ALA) and linoleic acid (LA), respectively. However, these conversions are believed to be insufficient for the growing infant and must be supplemented by diet. Human breast milk contains about 13–22 wt% of its total fatty acid content as PUFA, with the DHA content varying widely depending on the mother’s diet [10,11,12,13]. For example, in Japan, the average DHA content in breast milk is about 1% of total fatty acids while in Pakistan it is about 0.06% [14]. Studies in primate models have shown that maternal and neonatal diets deficient in ω-3 PUFAs result in altered brain and retinal fatty acid composition and are associated with impaired neural and visual function [15].

Fatty acid binding proteins (FABPs) are a family of 10 intracellular proteins that bind hydrophobic ligands including fatty acids [16,17]. Different members of the FABP family are expressed in the brain where they participate in the intracellular trafficking of different fatty acids [16]. Of these FABPs, brain fatty acid binding protein (B-FABP or FABP7) binds DHA with the highest affinity, although it can also bind other PUFAs such as AA [18]. FABP7 has well-established roles in brain development and has also been shown to be a key determinant of malignant glioma growth properties and prognosis [16,19,20,21,22]. It has been postulated that the relative levels of AA and DHA in brain may affect FABP7 expression [23]. A recent study suggests a link between FABP7, DHA and gene expression [24].

In this study, we explore the impact of DHA-rich maternal and weaning diets on brain fatty acid composition as well as FABP7 expression during the first six weeks of life. Although some reports have assessed the effect of feeding ω-3-rich diets to dams during pregnancy and lactation on brain fatty acid composition at embryonic and weanling stages [25,26], to our knowledge, there has been no follow-up at later developmental stages. The brains of three-week and six-week old rats correspond to that of 2–3 year old and 12–18 year old humans, respectively [7]. At these two developmental stages, the brains of rats and humans are thought to undergo fairly similar developmental processes [7]. Our model should therefore provide relevant information on brain fatty acid needs in humans at these two stages.

## 2. Experimental Section

### 2.1. Animals

Protocols involving animal use were approved by the University of Alberta Health Sciences Animal Care and Use Committee and were carried out following the Canadian Council on Animal Care guidelines and in compliance with the ARRIVE guidelines. Primiparous Sprague-Dawley rats (*n* = 20) were obtained from Charles River Laboratories (Montreal, Quebec, Canada) on day 14 of gestation. Dams were fed standard rat chow (Lab diet 5001; PMI Nutrition International, Brentwood, MO, USA) throughout gestation. Approximately 24 h before giving birth, dams were randomly allocated to one of two nutritionally adequate experimental diets. The composition of the diets, different only in fat composition, has been previously published [27]. The fatty acid composition of the control diet (Cnt, *n* = 12 dams) and the DHA-rich diet (ω-3, *n* = 8 dams) is described in Table 1. The fat content (20% w/w) and the polyunsaturated to saturated fatty acid (PUFA:SFA) ratio (0.5) did not differ between diets. All diets met the essential fatty acid requirements of the rodent. At birth, the litters were culled, leaving 10 pups per dam. Diets were fed *ad libitum* throughout the suckling period. Offspring were kept with their mothers until termination.

**Figure 1:**
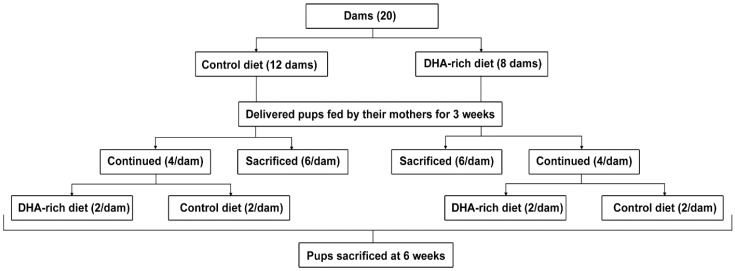
Outline of the study design. Dams were fed control (Cnt) or DHA-rich (ω-3) diets during lactation. Three weeks postnatally, dams and a portion of the pups were sacrificed and their brains were collected. Aliquots of dam breast milk and pup stomach content were collected to assess dietary effect on DHA content. The remaining pups were maintained on either control (Cnt) or DHA-rich (ω-3) diet for three more weeks until sacrificed and their brain samples were collected.

Three weeks postnatally, the dams and six pups/dam were weighed and sacrificed by CO_2_ exposure and subsequent cervical dislocation. Their brains were carefully excised, snap frozen in liquid nitrogen and kept at −80 °C until assayed for fatty acid composition and FABP7 expression. Pup stomach content was also collected and assayed for fatty acid composition as an indicator of dietary effect on maternal milk composition [28]. The remaining pups from each dam (*n* = 4) were randomly assigned either the same diet as the dam (*n* = 2/dam) or crossed over to the other diet (*n* = 2/dam). The pups were fed this diet for an additional three weeks. This design resulted in four groups of six-week old pups: Cnt/Cnt, ω-3/Cnt, Cnt/ω-3, and ω-3/ω-3, based on the respective dam diet and final pup diet (dam diet/pup diet). At six weeks, rats were sacrificed and brain samples were collected as described previously. An outline of the experimental design is presented in (Figure 1).

**Table 1:** Fatty acid composition of control (Cnt) and docosahexaenoic acid (DHA)-rich (ω-3) diets. Data are presented as % of total fatty acids ^a^.

Fatty Acid	Control Diet (Cnt)	DHA Diet (ω-3)
g/100 g of total fatty acids		
C14:0	0.1 ± 0.0	0.4 ± 0.0
C16:0	6.7 ± 0.3	6.2 ± 0.1
C16:1ω-7	0.2 ± 0.0	0.2 ± 0.1
C18:0	38.8 ± 1.2	40.6 ± 0.2
C18:1ω-9	29.0 ± 1.7	24.8 ± 0.3
C18:2ω-6 (LA)	21.2 ± 0.5	21.6 ± 0.0
C20:0	0.9 ± 0.0	0.9 ± 0.0
C18:3ω-3 (ALA)	1.7 ± 0.1	3.3 ± 0.1
C20:3ω-6	0.4 ± 0.1	0.4 ± 0.1
C20:4ω-6 (AA)	0.4 ± 0.0	0.4 ± 0.0
C22:6ω-3 (DHA)	0	0.9 ± 0.1

^a^ Analysis by GLC of *n* = 2 batches, mean ± standard error of the mean (SEM); AA, arachidonic acid; ALA, α-linolenic acid; DHA, docosahexaenoic acid; ω, omega.

### 2.2. Brain Phospholipids Fatty Acid Composition

Brain lipids were extracted by a modified Folch method and total phospholipids were separated on silica G plates as previously described [29]. The bands were visualized with 8-anilino-1-naphthalenesulfonic acid under UV light. Appropriate standards were used for comparison. Fatty acid methyl esters were then prepared from the scraped silica bands and separated by automated gas liquid chromatography (Agilent Model 7890A, Agilent Technologies, Mississauga, ON, Canada) using a 100 m CP-Sil 88 fused capillary column (Agilent Technologies) with peaks identified by comparison with standards (NuChek Prep, Elysian, MN, USA). A total of 20 fatty acids were assessed and their values were expressed as g/100 g of total fatty acids.

### 2.3. Western Blot Analysis

Brain tissue was homogenized in modified RIPA buffer [50 mM Tris-HCl pH 7.0, 150 mM NaCl, 0.5% sodium deoxycholate, 1% NP40, 0.1% SDS, 1 mM sodium fluoride, 1× Complete Protease Inhibitor (Roche Diagnostics, Laval, Canada)]. Brain lysates (50 μg protein per lane) were electrophoresed in 12.5% SDS-polyacrylamide gels then transferred to nitrocellulose membranes and immunostained with rabbit anti-FABP7 antibody (1:500 dilution) [30] or goat anti-actin antibody (1:100,000) (Sigma-Aldrich, Oakville, Canada). Primary antibodies were detected with horseradish peroxidase-conjugated secondary antibodies (Jackson ImmunoResearch Laboratories Inc., Burlington, Canada) using the ECL detection system (GE Healthcare, Mississauga, Canada).

### 2.4. Statistical Analysis

Data are reported as mean ± standard error of the mean (SEM). In analyzing pup data, every dam was represented by one pup and pups were treated as individual experimental units. One-way analysis of variance, Duncan’s multiple range *post hoc* test (4 groups) and Student’s unpaired *t*-test (2 groups) were used to assess the significance of differences between groups. Where data did not conform to normality, Kruskal-Wallis one way analysis of variance on ranks and Dunn’s test were used. Microsoft Excel (Microsoft, Redmond, WA, USA) and SigmaPlot 12.0 (Systat software, Inc. Chicago, IL, USA) were used in the statistical analysis of data. The level of significance was set at *p* < 0.05.

## 3. Results

### 3.1. Effect of a DHA-Rich Diet on Dam and Pup Brain Fatty Acid Composition

#### 3.1.1. Dam Brain Fatty Acid Composition: DHA-Rich Diet Does Not Alter AA and DHA Levels

Dams were divided into two groups: those fed a control diet (Cnt) consisting of 1.7% total ω-3 PUFA (no added DHA), and those fed a DHA-rich diet (ω-3) consisting of 0.9% DHA with a total ω-3 PUFA content of 4.2% (Figure 1; Table 1). The ω-6:ω-3 ratios of the control and DHA-rich diets were 13.3:1 and 5.3:1, respectively. Dams were fed the diets until they were sacrificed 21 days after delivery. Litter size and body weights of Cnt and ω-3 dams did not differ significantly with average values being 323 ± 12.0 g and 299 ± 4.23 g at termination, respectively.

There were no significant differences in total saturated fatty acids (SFA), total monounsaturated fatty acids (MUFA), and total PUFA between the Cnt and ω-3 dam brains (Table 2) although a borderline (*p* = 0.053) reduction (by 8.2%) in ω-6 PUFA was observed in ω-3 dam brains compared to controls. Of note, LA (C18:2ω-6) was significantly increased (by 9.4%) whereas adrenic acid (C22:4ω-6) was significantly decreased (by 55.6%) in ω-3 dam brains. C22:4ω-6, a major PUFA in myelin, is formed from C20:4ω-6 by a 2-carbon chain elongation.

The ratio of C18:2ω-6:C20:4ω-6 was significantly increased (by 17.2%) in ω-3 dam brains compared to controls, indicating reduced conversion of LA to AA in dams fed a DHA-rich diet. A DHA diet also reduced the ω-6:ω-3 PUFA ratio by 12.5% (*p* = 0.046), and there was a trend towards a reduced AA:DHA ratio (by 12.3%, *p* = 0.065). Thus, the most dramatic effect of a DHA-rich diet on dam brain fatty acid composition is about a two-fold reduction in C22:4ω-6 levels, with no change in ω-3 PUFA levels and a borderline decrease in overall ω-6 PUFA levels.

#### 3.1.2. Three-Week Old Pup Brain Fatty Acid Composition: DHA-Rich Diet Increases DHA and Decreases AA Content

At three weeks, pups weaned from Cnt or ω-3 dams showed no significant difference in body weight, with weights being 50.5 ± 0.74 g and 50.7 ± 1.3 g, respectively. DHA content was higher in milk samples collected from ω-3 dams compared to controls (1.43% ± 0.12% *vs.* 0.19% ± 0.02%, *p* < 0.05) with no change in AA content (2.9% ± 0.6% *vs.* 2.5% ± 0.3%, full composition not shown). The stomach contents of three-week pups were also collected as these should reflect the fatty acid composition of dams’ milk. The increase in DHA observed in the breast milk of dams fed a DHA-rich diet was mirrored by pup stomach content with a DHA content of 1.1% ± 0.03% in ω-3 samples compared to 0.24% ± 0.18% in Cnt samples.

**Table 2:** Fatty acid composition of total phospholipids isolated from brains of dams fed either control (Cnt) or DHA-rich (ω-3) diet.

Fatty Acids	Cnt (*n* = 9)	ω-3 (*n* = 4)	*p*-Value *
	% of total fatty acids		
14:0	0.22 ± 0.07	0.29 ± 0.16	0.63
15:0	0.45 ± 0.15	0.52 ± 0.29	0.82
16:0	22.2 ± 0.15	22.9 ± 0.17 *	0.03
16:1ω-9	0.49 ± 0.03	0.51 ± 0.03	0.63
18:0	25.0 ± 0.22	25.1 ± 0.68	0.94
18:1ω-9	17.7 ± 0.25	17.5 ± 0.65	0.81
18:1c11	4.5 ± 0.38	4.4 ± 0.59	0.88
18:2ω-6	0.70 ± 0.01	0.77 ± 0.02 *	0.004
20:0	0.37 ± 0.02	0.37 ± 0.04	0.94
18:3ω-3	1.1 ± 0.05	1.1 ± 0.07	0.74
20:2ω-6	0.09 ± 0.008	0.09 ± 0.003	0.64
20:3ω-6	0.81 ± 0.05	0.77 ± 0.06	0.62
20:4ω-6	8.9 ± 0.25	8.3 ± 0.11	0.12
24:0	1.2 ± 0.07	1.2 ± 0.15	0.86
24:1ω-9	2.7 ± 0.12	2.5 ± 0.06	0.14
22:4ω-6	0.53 ± 0.04	0.24 ± 0.09 *	0.004
22:5ω-6	0.05 ± 0.008	0.06 ± 0.01	0.32
20:5ω-3	0.73 ± 0.05	0.73 ± 0.1	0.99
22:5ω-3	0.12 ± 0.01	0.16 ± 0.01	0.07
22:6ω-3	12.0 ± 0.28	12.6 ± 0.30	0.19
Total MUFA	25.3 ± 0.32	24.8 ± 0.07	0.16
Total SFA	49.5 ± 0.33	50.3 ± 0.44	0.18
ω-3 PUFA	13.9 ± 0.27	14.6 ± 0.36	0.19
ω-6 PUFA	11.1 ± 0.27	10.2 ± 0.13	0.05
Total PUFA	25.1 ± 0.34	24.8 ± 0.35	0.65
18:2ω-6:20:4ω-6	0.08 ± 0.003	0.09 ± 0.003 *	0.02
ω-6:ω-3 PUFA	0.8 ± 0.03	0.7 ± 0.02 *	0.046

Data are presented as mean ± standard error of the mean (SEM). * indicates significant difference between the two groups. MUFA: monounsaturated fatty acid; SFA: saturated fatty acid; PUFA: polyunsaturated fatty acids. * *p* < 0.05 indicates significant difference between the fatty acid levels in brain phospholipids of dams fed control diet *versus* ω-3 diet. Two-tailed unpaired *t*-test.

The brain composition of three-week old pups showed no change in terms of total SFA, total MUFA, and total PUFA content with DHA-rich diet. However, significant differences were observed within the PUFA category, with ω-6 PUFAs decreasing by 9.9%, and ω-3 PUFAs increasing by 15.3% in three-week old pups whose dams were fed a DHA-rich diet (Table 3). For ω-6 PUFA, C22:4 and C20:4 were significantly decreased by 64.4% and 9.0%, respectively, in keeping with the results observed for dams. As well, the C18:2ω-6:C20:4ω-6 ratio was significantly increased (by 22.1%). Thus, 3-week pup brains mimics dam brains in that conversion of LA to AA is decreased under DHA-rich conditions.

The increase in ω-3 PUFA observed in the brains of pups from dams fed a DHA-rich diet was mainly due to a 13.6% increase in C22:6 (Table 3). These changes in ω-6 and ω-3 PUFAs resulted in a 21.9% decrease in the ω-6:ω-3 PUFA ratio and a 20.1% decrease in the AA:DHA ratio (Table 3). We also observed a 13.4% decrease in the MUFA, C24:1ω-9 (nervonic acid), in pup brains as a consequence of high levels of DHA in the maternal diet (Table 3). The effect of a DHA-rich diet on C24:1ω-9 in pup brains is of potential significance as nervonic acid is a major component of myelin. These results demonstrate that pup brain fatty acid composition prior to weaning is highly dependent on dam diet, with significant increases observed in DHA content, as well as decreases in 22:4ω-6, C20:4ω-6 and 24:1ω-9 content.

**Table 3:** Fatty acid composition of total phospholipids isolated from brains of three-week old pups weaned from dams fed either control (Cnt) or DHA-rich (ω-3) diet.

Fatty Acids	Cnt (*n* = 15)	ω-3 (*n* = 9)	* *p*-Value
	% of total fatty acids		
14:0	0.42 ± 0.01	0.37 ± 0.02	0.05
15:0	0.77 ± 0.09	0.61 ± 0.12	0.29
16:0	27.5 ± 0.23	26.1 ± 0.69	0.08
16:1ω-9	1.1 ± 0.03	1.0 ± 0.04	0.20
18:0	23.7 ± 0.16	23.7 ± 0.3	0.99
18:1ω-9	13.3 ± 0.17	14.4 ± 0.79	0.22
18:1c11	2.7 ± 0.09	2.9 ± 0.20	0.16
18:2ω-6	1.1 ± 0.03	1.2 ± 0.04	0.07
20:0	0.25 ± 0.02	0.36 ± 0.08	0.23
18:3ω-3	0.38 ± 0.03	0.58 ± 0.15	0.21
20:2ω-6	0.18 ± 0.008	0.20 ± 0.02	0.19
20:3ω-6	0.70 ± 0.03	0.99 ± 0.14	0.07
20:4ω-6	11.6 ± 0.16	10.6 ± 0.43 *	0.04
24:0	0.31 ± 0.04	0.53 ± 0.16	0.19
24:1ω-9	3.2 ± 0.04	2.8 ± 0.04 *	<0.0001
22:4ω-6	1.4 ± 0.03	0.49 ± 0.02 *	<0.0001
22:5ω-6	0.03 ± 0.003	0.05 ± 0.01	0.14
20:5ω-3	0.39 ± 0.04	0.52 ± 0.14	0.42
22:5ω-3	0.14 ± 0.006	0.14 ± 0.01	0.88
22:6ω-3	10.8 ± 0.23	12.3 ± 0.43 *	0.003
Total MUFA	20.3 ± 0.23	21.2 ± 0.98	0.40
Total SFA	52.9 ± 0.31	51.6 ± 0.78	0.14
ω-3 PUFA	11.7 ± 0.21	13.5 ± 0.27 *	<0.0001
ω-6 PUFA	14.9 ± 0.13	13.5 ± 0.28 *	<0.0001
Total PUFA	26.7 ± 0.31	27.0 ± 0.44	0.56
ω-6 PUFA:ω-3 PUFA	1.3 ± 0.02	0.99 ± 0.02 *	<0.0001
20:4ω-6:22:6ω-3	1.1 ± 0.02	0.86 ± 0.02 *	<0.0001

Data are presented as mean ± standard error of the mean (SEM). MUFA: monounsaturated fatty acid; SFA: saturated fatty acid; PUFA: polyunsaturated fatty acids. One to two pups per dam were used for the fatty acid analyses. * *p* < 0.05 indicates significant difference between the fatty acid levels in brain phospholipids of three-week old pups from dams fed control diet *versus* ω-3 diet. Two-tailed unpaired *t*-test.

#### 3.1.3. Six-Week Old Pup Brain Fatty Acid Composition: DHA-Rich Diet Maintains Elevated DHA Content after Pups Are Transferred to a Cnt Diet

**Figure 2:**
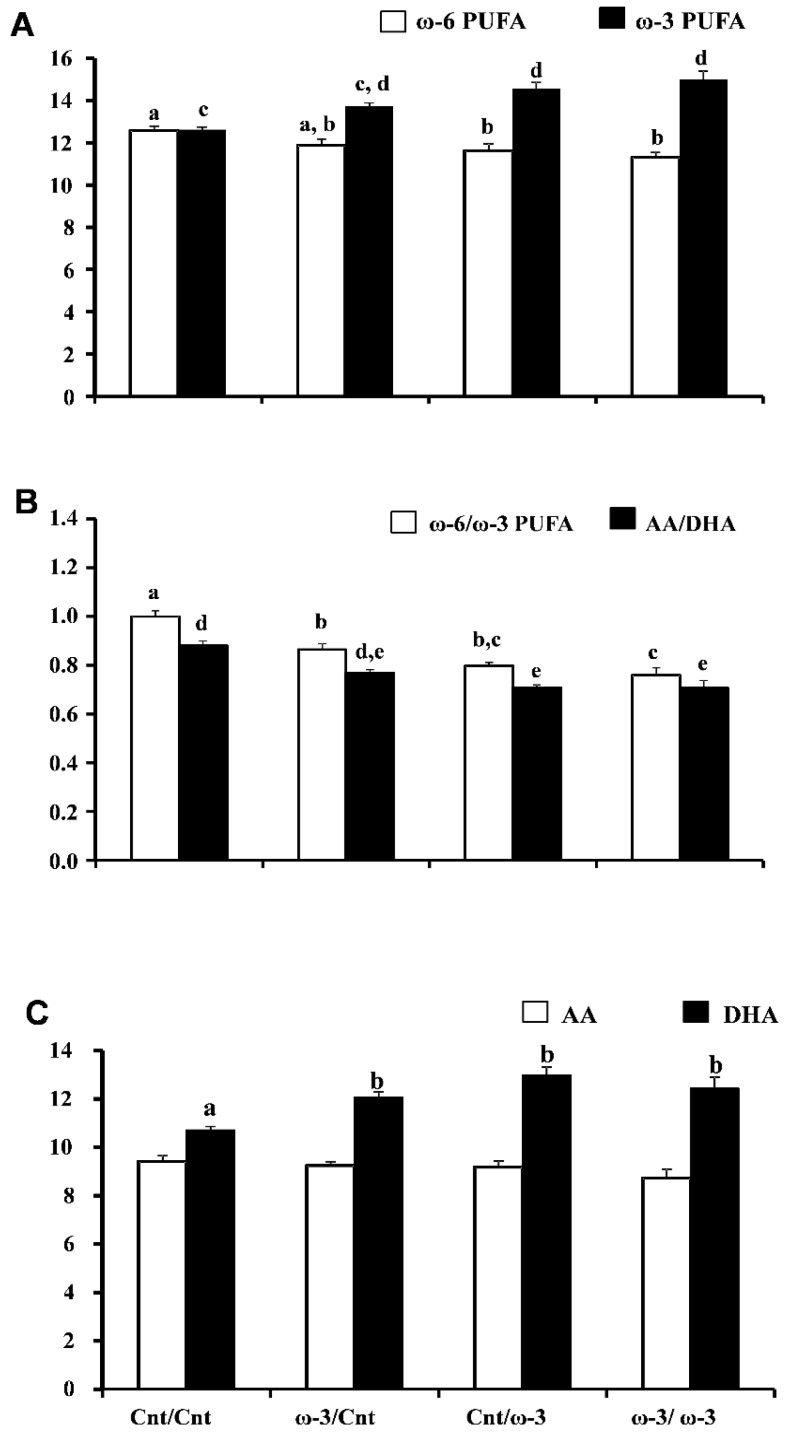
Effect of docosahexaenoic acid (DHA)-rich (ω-3) diet on brain long chain polyunsaturated fatty acids (PUFA) content in 6-week pups. The four sets of columns represent the Cnt/Cnt, ω-3/Cnt, Cnt/ω-3, and ω-3/ω-3 groups. Panel **A** shows ω-6 PUFA content and ω-3 PUFA content. Panel **B** shows ω-6:ω-3 PUFA and arachidonic acid (AA):DHA ratios. Panel **C** shows AA content and DHA content. Different letters indicate that groups are significantly different. Differences were assessed for significance using one-way analysis of variance followed by Duncan’s multiple range *post hoc* test for normally distributed data. Where the ranked data did not conform to normality, Kruskal-Wallis one way analysis of variance on ranks and Dunn’s test were used; *p* < 0.05. Numbers of pups included in the fatty acid assay at six weeks were 10, 7, 8 and 8 for the Cnt/Cnt, ω-3/Cnt, Cnt/ω-3, and ω-3/ω-3 groups, respectively. In all dietary groups, one pup per dam was used for the fatty acid analyses.

In six week pups, there were no significant changes in the body weights among the four dietary groups, with values being 159.5 ± 4.1, 163.3 ± 4.5, 158.2 ± 4.8, 156.5 ± 4.7 g, for Cnt/Cnt, ω-3/Cnt, Cnt/ω-3, and ω-3/ω-3 groups, respectively. Similarly, total SFA, total MUFA and total PUFA were not significantly different among the four groups (Table 4). Although total PUFA content was not significantly different, statistically significant increases in ω-3 PUFA were observed when comparing Cnt/ω-3 and ω-3/ω-3 treatment groups to the Cnt/Cnt group, with increases of 15.5% and 18.9%, respectively. The 9% increase observed upon comparing the ω-3 PUFA content in the ω-3/Cnt group to that of the Cnt/Cnt group was not statistically significant (Figure 2A and Table 4). A trend towards reduced ω-6 PUFA was apparent in ω-3/Cnt (5.7%) compared to Cnt/Cnt group, with significant reductions observed in the Cnt/ω-3 (7.6%) and ω-3/ω-3 (10.1%) groups (Figure 2A and Table 4).

**Table 4:** Fatty acid composition of total phospholipids isolated from brains of six-week old pups fed control diet (Cnt) or DHA-rich (ω-3) diet for three weeks after being weaned from dams that were fed either Cnt or ω-3 diets.

Fatty Acids	Treatment Groups (Dam diet/Pup Diet)			
	Cnt/Cnt (*n* = 10)	ω-3/Cnt (*n* = 7)	Cnt/ω-3 (*n* = 8)	ω-3/ω-3 (*n* = 8)
	% of Total Fatty Acids			
14:0	0.17 ± 0.006	0.17 ± 0.004	0.17 ± 0.004	0.16 ± 0.005
15:0	0.61 ± 0.21	0.77 ± 0.27	0.74 ± 0.22	1.1 ± 0.17
16:0	22.9 ± 0.28	24.5 ± 1.1	23.6 ± 0.34	21.8 ± 0.72
16:1ω-9	0.48 ± 0.01	0.55 ± 0.03	0.50 ± 0.02	0.48 ± 0.02
18:0	25.2 ± 0.46	24.5 ± 0.89	25.4 ± 0.49	23.7 ± 0.62
18:1ω-9	16.5 ± 0.39	16.4 ± 0.74	16.0 ± 0.21	18.3 ± 0.86
18:1c11	4.0 ± 0.31	3.2 ± 0.32	3.3 ± 0.34	3.1 ± 0.21
18:2ω-6	0.78 ± 0.04 a	0.80 ± 0.03 a	0.82 ± 0.03 a,b	0.94 ± 0.06 b
20:0	0.48 ± 0.05 a,b	0.45 ± 0.06 a,b	0.38 ± 0.01 a	0.58 ± 0.09 b
18:3ω-3	0.99 ± 0.12	0.78 ± 0.11	0.78 ± 0.05	1.4 ± 0.28
20:2ω-6	0.16 ± 0.02	0.13 ± 0.008	0.15 ± 0.009	0.19 ± 0.03
20:3ω-6	0.87 ± 0.07 a,b	0.83 ± 0.11 a,b	0.77 ± 0.02 a	1.1 ± 0.09 b
20:4ω-6	9.4 ± 0.23	9.3 ± 0.15	9.2 ± 0.25	8.7 ± 0.35
24:0	1.1 ± 0.11	0.95 ± 0.19	0.79 ± 0.03	1.3 ± 0.27
24:1ω-9	3.1 ± 0.06 a	2.7 ± 0.04 b	2.7 ± 0.1 b	2.7 ± 0.08 b
22:4ω-6	1.3 ± 0.08 a	0.81 ± 0.32 a,b	0.68 ± 0.08 a,b	0.28 ± 0.03 b
22:5ω-6	0.03 ± 0.003	0.04 ± 0.005	0.03 ± 0.001	0.05 ± 0.01
22:5ω-3	0.11 ± 0.007 a	0.13 ± 0.009 a,b	0.15 ± 0.02 a,b	0.16 ± 0.009 b
22:6ω-3	10.7 ± 0.15 a	12.1 ± 0.25 b	13.0 ± 0.35 b	12.4 ± 0.45 b
Total MUFA	24.1 ± 0.37 a,c	22.9 ± 0.56 a,b	22.4 ± 0.44 b	24.7 ± 0.75 c
Total SFA	50.4 ± 0.36	51.4 ± 0.42	51.1 ± 0.58	48.7 ± 0.90
ω-3 PUFA	12.6 ± 0.13 a	13.7 ± 0.14 a,b	14.6 ± 0.29 b	15.0 ± 0.40 b
ω-6 PUFA	12.6 ± 0.21 a	11.9 ± 0.31 a,b	11.6 ± 0.32 b	11.3 ± 0.23 b
Total PUFA	25.2 ± 0.19	25.6 ± 0.34	26.2 ± 0.58	26.3 ± 0.34
18:2ω-6:20:4ω-6	0.08 ± 0.006 a	0.09 ± 0.005 a,b	0.09 ± 0.004 a,b	0.11 ± 0.01 b
ω-6 PUFA:ω-3 PUFA	0.99 ± 0.02 a	0.87 ± 0.02 b	0.79 ± 0.01 b,c	0.76 ± 0.03 c
20:4ω-6:22:6ω-3	0.88 ± 0.02 a	0.77 ± 0.01 a,b	0.71 ± 0.01 b	0.71 ± 0.03 b

Data are presented as mean ± standard error of the mean (SEM). Differences were assessed for significance using one-way analysis of variance followed by Duncan’s multiple range post hoc test for normally distributed data. Where the ranked data did not conform to normality, Kruskal-Wallis one way analysis of variance on ranks and Dunn’s test were used. Significant difference (*p* < 0.05) between the different treatment groups is indicated by different letters across a row. One pup per dam was included in the analyses of the fatty acid composition for each group.

A significant reduction in the ω-6:ω-3 PUFA ratio was observed in ω-3/Cnt (13.5%), Cnt/ω-3 (20.1%), and ω-3/ω-3 (24.0%) compared to Cnt/Cnt pups (Figure 2B and Table 4). The ratio of AA:DHA was reflective of the overall change in ω-6:ω-3 PUFA ratio and was also decreased in ω-3/Cnt (12.6%), Cnt/ω-3 (19.5%), and ω-3/ω-3 (19.7%) compared to Cnt/Cnt pups; however, statistical significance was not attained when ω-3/Cnt pups were compared to Cnt/Cnt pups (Figure 2B and Table 4).

The increase in ω-3 PUFA content observed in six week pups was primarily due to increased DHA content (Figure 2C and Table 4). DHA was significantly increased in ω-3/Cnt, Cnt/ω-3, and ω-3/ω-3, by 12.5%, 21.2%, and 16.2%, respectively, when compared to Cnt/Cnt pups (Figure 2C and Table 4). C22:5ω-3 (EPA), a precursor of DHA that is normally found at very low levels in brain, was significantly increased by 51.2% in ω-3/ω-3 compared to Cnt/Cnt pups, with a trend towards increased EPA levels observed for ω-3/Cnt (21.9% increase) and Cnt/ω-3 pups (37.2% increase) (Table 4).

As previously noted for dam and three-week pup brains, the ω-6 PUFA, C22:4 was significantly decreased (by 79%) in ω-3/ω-3 brains, with a non-significant trend towards decreased levels observed in ω-3/Cnt (by 38.6%) and Cnt/ω-3 (by 48.6%) pups, compared to Cnt/Cnt pups (Table 4). LA (C18:2ω-6) was increased by 21.2% and 17.3% in the brains of ω-3/ω-3 pups compared to Cnt/Cnt and ω-3/Cnt pups, respectively, with the C18:2ω-6:C20:4ω-6 ratio being significantly increased in ω-3/ω-3 pups (by 32.2%) compared the Cnt/Cnt pups (Table 4). A trend towards an increased C18:2ω-6:C20:4ω-6 ratio was also observed in ω-3/Cnt (by 4.4%) and Cnt/ω-3 (by 12.9%) pups compared to Cnt/Cnt pups (Table 4).

The MUFA, C24:1ω-9, was significantly decreased in the brains of pups exposed to a DHA-rich diet, with percentage reductions of 11.7%, 13.1%, and 10.5% in ω-3/Cnt, Cnt/ω-3, and ω-3/ω-3 pups, respectively, compared to Cnt/Cnt pups (Table 4). Thus, our diet crossover experiment indicates that DHA feeding during lactation maintains a brain environment that favors ω-3 PUFA enrichment even after the pups are transferred to a low-DHA diet post weaning. Our data also show that continued feeding of a DHA-rich diet is needed for inhibition of the ω-6 metabolic conversion of LA to AA.

### 3.2. FABP7 Expression in Three-Week and Six-Week Pups

**Figure 3:**
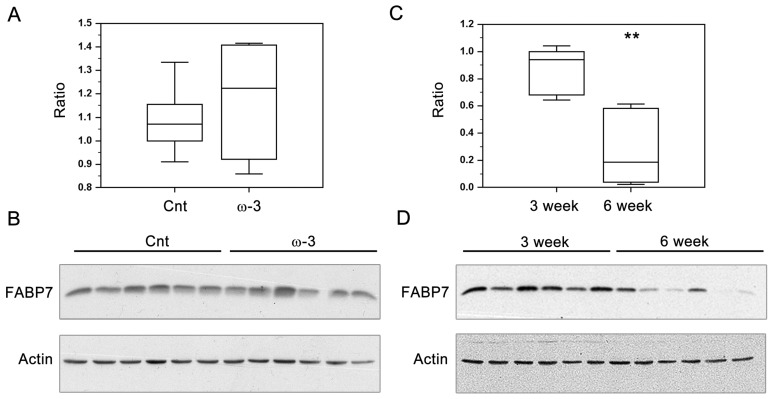
Effect of docosahexaenoic acid (DHA)-rich (ω-3) diet and age on brain fatty acid binding protein expression (FABP7). Box-plots represent band intensities of FABP7/actin (Y-axis) in the brains of three-week old pups fed a control diet (Cnt, *n* = 6; one pup per dam) or DHA-rich (ω-3, *n* = 6; one pup per dam) diet (**A**), and in the brains of three-week old pups (*n* = 6; one pup per dam) and six-week (*n* = 6; one pup per dam) fed a Cnt diet (**C**). Panels B and D are Western blots showing FABP7 and actin levels in the brains of three-week old pups fed control and DHA-rich diets (**B**), and three and six-week old pups fed a Cnt diet (**D**). ** indicates *p* < 0.01. Differences were assessed for significance using two-tailed unpaired *t*-test.

**Figure 4:**
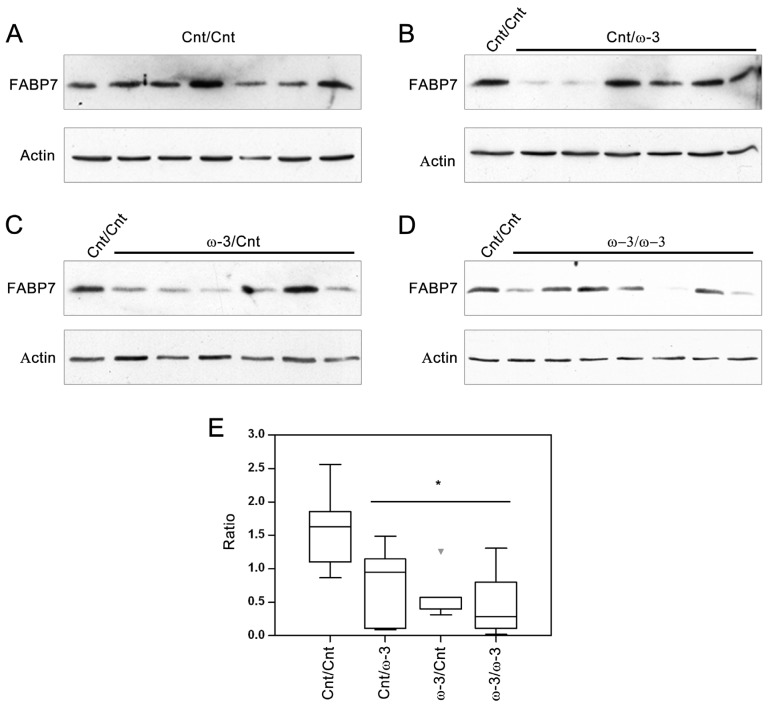
Effect of a docosahexaenoic acid (DHA)-rich (ω-3) diet on brain fatty acid binding protein expression (FABP7) at six weeks. Panels **A**, **B**, **C** and **D** are western blots showing FABP7 and actin levels in the brains of six-week old Cnt/Cnt (*n* = 7; one pup per dam), Cnt/ω-3 (*n* = 6; one pup per dam), ω-3/Cnt (*n* = 6; one pup per dam), and ω-3/ω-3 (*n* = 6; one pup per dam) pups, respectively. Panel **E** represents a Box-Plot of band intensities of FABP7 (Y-axis, normalized to actin and a Cnt/Cnt sample that was loaded in lane 1 of all gels to normalize for any differences in gel handling and electro-blotting) in different dietary groups. * indicates significant difference (*p* < 0.05) from the Cnt/Cnt with groups under the line being statistically equivalent.

We next assessed the relationship between DHA intake and FABP7 protein levels as it has previously been postulated that the increase in the AA:DHA ratio observed in malignant glioma tumor tissue might be associated with changes in FABP7 expression [23]. Dam diet had no significant effect on FABP7 protein levels in three-week pups (Figure 3A, B). As expected, a significant reduction (69%) in FABP7 expression was observed in six-week pups compared to three-week pups fed a Cnt diet (Figure 3C, D). However, at six weeks, when FABP7 levels are generally low, a DHA-rich diet did have an effect on FABP7 levels, with significant reductions observed whether DHA was provided through the dam or as a three-week long dietary supplement starting when the pups were weaned. Percent reductions were 65%, 50%, and 70% in ω-3/Cnt, Cnt/ω-3 and ω-3/ω-3 pups compared to Cnt/Cnt pups, respectively (Figure 4A–E).

## 4. Discussion

DHA-induced improvement in brain function is believed to be due to its modulation of synaptic proteins and overall activity [31]. Our results indicate that the developing brain readily incorporates DHA supplied during toddler/juvenile stages since the brains of six-week old rats are developmentally equivalent to 12–18 year-old human brains [32]. Furthermore, early introduction of DHA (during lactation) maintains high DHA levels in the brain even after the pups are switched to low-DHA weaning diet. Importantly, boosting brain DHA levels is still achievable through direct dietary supply at weaning in cases where DHA was not provided during suckling. Finally, we report that DHA down-regulates the expression of FABP7, a key factor associated with neural proliferation and differentiation [33,34]; however, this effect is only apparent later in brain development.

Diets were designed to provide an adequate supply of ω-6 (including AA) and ω-3 PUFA in the absence (Cnt) or presence of DHA (ω-3). Our dietary ratios of total ω-6 to total ω-3 PUFA are within the previously reported range in human breast milk samples, as well as milk fatty acid profiles of rats [10,12,35]. We observed the following effects of DHA feeding on brain lipid composition at the toddler and juvenile stages: (i) increased brain total ω-3 PUFA content (especially DHA), (ii) reduced brain total ω-6 PUFAs, (iii) reduced ω-6:ω-3 ratio and AA:DHA ratio, and (iv) reduced brain adrenic (C22:4ω-6) and nervonic acid (C24:1ω-9) content.

It is well known that adult brains are more resistant than juvenile brains to diet-induced changes in fatty acid composition [36]. Minor increases in brain DHA and total ω-3 PUFA content (~5%) with concomitant decreases in AA and total ω-6 PUFA (5–6%) have previously been reported in adult rats fed DHA-rich diet for eight weeks [37]. These observations are consistent with our results in lactating dams that were fed a DHA-rich diet for three weeks. We did observe increases in LA (C18:2ω-6) and in the LA:AA ratio (C18:2ω-6:C20:4ω-6), along with a decrease in C22:4ω-6 (adrenic acid), in the brains of dams fed a DHA-rich diet. These changes likely indicate DHA-mediated inhibition of LA metabolism through ∆-6 and ∆-5 desaturases or inhibition of AA elongation, as previously reported [38,39,40]. The lack of effect on AA levels is likely due to the adequate supply of AA in the DHA-rich (ω-3) diet.

In keeping with previous work, we observed more pronounced DHA-rich diet-induced changes in brain fatty acid composition at three weeks [26,41]. Human infant brains have been reported to be similarly susceptible to changes in dietary fatty acids, with higher (39%) brain DHA accumulation in six-month old breast-fed infants compared to infants fed formula that did not contain AA or DHA [42]. Our results indicate that the DHA-rich diet increases total ω-3 PUFA (mainly DHA) by 14.7% and decreases total ω-6 PUFA (mainly AA and C22:4ω-6) and ω-6:ω-3 ratio by 12.8% and 24%, respectively. Since the brain of a three-week old rat is at a comparable stage as that of a human toddler (2-3 years), our results suggest that DHA accretion in human brain may well extend beyond 6 months. As with the dams, decreases in LA metabolic products (C22:4ω-6 and AA), together with an increase in the LA:AA ratio, were observed at three weeks, indicating inhibition of ∆-6 and ∆-5 desaturases by DHA [43,44,45,46].

At six weeks, changes in brain fatty acid composition were most marked in pups born to Cnt dams and fed a DHA-rich diet (Cnt/ω-3) or born to ω-3 dams and maintained on a DHA-rich diet (ω-3/ω-3). There is clear indication of inhibition of the ω-6 PUFA desaturation and elongation pathway in these pups. Unlike changes in ω-6 PUFA which were readily reversible, increases in the levels of DHA in six-week old pup brains were not reversed when DHA was discontinued. In fact, brain DHA levels in ω-3/Cnt pups showed increases that were equivalent to pups fed a DHA-rich diet for three weeks post-weaning (Cnt/ω-3) or those exposed to DHA from birth up to six postnatal weeks (ω-3/ω-3 group). In comparison, DHA-induced decreases in ω-6 PUFA were readily reversible and disappeared when DHA was discontinued (ω-3/Cnt group). It will be important to determine whether the effect of a maternal DHA-rich diet on pup brain DHA levels can be extended past six weeks.

Levels of nervonic acid (C24:1ω-9) in three-week and six-week pup brains were significantly reduced by increased levels of DHA in the diet. Nervonic acid is the major very long chain fatty acid found in sphingomyelin, one of the main components of myelin [47,48]. Although studies show that there is postnatal accretion of nervonic acid in sphingomyelin, there are no systematic reports assessing the effect of a DHA-rich diet on myelination [6,49]. Interestingly, a diet high in DHA results in longer latencies of the auditory startle response (a functional indicator of myelination) [50]. In contrast to MUFA such as nervonic acid, PUFA content in myelin phospholipids is low, consisting of 1/6 to 1/3 of the PUFA content of gray matter phospholipid [48]. Adrenic acid (C22:4ω-6) is a major PUFA of myelin [6]. As our DHA-rich diet also significantly decreased adrenic acid levels in the brains of three-week and six-week pups, it will be important to carry out follow-up studies on the effect of a DHA-rich diet on the myelination of juvenile brain.

The mammalian brain has elevated levels of DHA and AA compared to other tissues, with the DHA:AA ratio increasing as a function of brain maturation [6,9]. Analysis of human brain at different stages has revealed different ratios of DHA:AA in the different phospholipid classes, with phosphotidylserine having the highest DHA:AA ratio and phosphotidylcholine having the lowest DHA:AA ratio [6]. The DHA:AA ratio in phosphotidylethanolamines changes over the course of brain maturation, from <1:1 to >1:1. It has been estimated that phosphotidylserine and phosphotidylethanolamine contain ~92% of the esterified DHA in total brain phospholipids of one-week old rat pups [8].

Different regions of the brain as well as the different phospholipid classes show different susceptibilities to diet-induced changes in fatty acid composition. For example, the DHA-rich frontal cortex appears to be particularly sensitive to ω-3 PUFA deficiency [51,52]. Phosphatidylethanolamines prepared from neuronal cells isolated from frontal cortex, cerebellum and hippocampus of pups whose dams were fed various diets during lactation showed differential accretion of DHA and AA over time depending on diet [53]. For example, one-week to three-week old pups born from dams fed either a diet with an LA to ALA ratio of 4:1 or a DHA-supplemented (0.8 g/100 g fat) diet showed steady increases in DHA levels, especially in the cerebellum. Extending the dam diet to weaned pups for an additional three weeks resulted in further accretion of DHA in the cerebellum, but not in the frontal lobe or hippocampus [53]. In comparison, DHA supplementation (0.8 g/100 g fat) had no effect on DHA levels in phosphotidylcholine in all three regions tested, although six-week old pups did show increased accretion of DHA in this phospholipid subclass in the frontal lobe and to a lesser extent in cerebellum [53]. Thus, results from the phosphotidylethanolamine analysis are in general agreement with our results, with the exception that we did not observe a further increase in DHA levels in whole brain phospholipids at six weeks compared to three weeks in pups fed a continuous DHA-rich diet for six weeks.

We have previously noted associations between the AA:DHA ratio and FABP7 in normal brain and brain tumors [23]. For example, FABP7 levels are high during normal brain development when the AA:DHA ratio is relatively high [8,9,19]. FABP7 expression decreases from birth onwards, a period that coincides with high brain DHA accumulation and a lower AA:DHA ratio [9,19]. Furthermore, the AA:DHA ratio in human malignant glioma tumors is increased compared to that of normal brain [54], with FABP7 expression also up-regulated in these tumors [21,55]. During brain development, FABP7 is expressed in radial glial cells, neural stem/progenitor cells that have self-renewal capacity and can differentiate into both neuronal and glial cells [19,56,57]. Radial glial cells form the fiber network along which neurons migrate in developing brain. Although radial glial cells are primarily found in developing brain, these cells are also retained in the centers of the brain that undergo neurogenesis in the adult [57]. *In vitro* binding studies indicate that FABP7 has a special affinity for PUFAs, including DHA and AA [18,58].

In this study, we tested the hypothesis that increased FABP7 levels are associated with a high AA:DHA ratio in a normally developing brain. Interestingly, we found that boosting brain DHA levels (thus decreasing the AA:DHA ratio) was associated with significant reductions in FABP7 levels at six weeks. The correlation between high levels of FABP7 and AA suggests a role for FABP7/AA in processes related to radial glial cell function such as formation of the fiber network that guide neuronal migration. Thus, there may be a reduced need for FABP7/AA-mediated events in the brains of six-week old pups exposed to a DHA-rich diet. In general agreement with our observation that changes in FABP7 levels were noted at six weeks but not at three weeks, Pelerin *et al.* reported little if any change in *FABP7* RNA levels in the cortex and microvessels of P14 pups whose dams were fed a DHA-supplemented diet [59]. Brains from older pups were not analyzed by these investigators.

Studies involving humans and pigs have shown that brain DHA content increases postnatally (up to eight years and 14 weeks, respectively) while brain AA plateaus or decreases postnatally [6,9]. Similar patterns have been observed in rats at ED17, ED20, and P8, with AA:DHA ratios of ~2 and ~1 observed in total brain lipids at E17 and P8, respectively [8]. While we didn’t observe an increase in DHA content from three weeks to six weeks, there was a decrease in AA content during this period (by 19%) (Supplemental Figure S1), resulting in an overall decrease in the AA:DHA ratio as the brain assumes higher levels of structural and functional maturation. Along with this change in the AA:DHA ratio, we observed a significant reduction (69%) in brain FABP7 levels from three weeks to six weeks. Thus, we propose that a DHA-rich diet during lactation and/or weaning may enhance or accelerate brain maturation, as suggested by the observed: (i) increase in ω-3 PUFA; (ii) decrease in ω-6 PUFA; (iii) decrease in ω-6:ω-3 PUFA (and AA:DHA) ratio; and (iv) decrease in FABP7 protein levels.

## 5. Conclusions

We have examined the impact of DHA-rich maternal and weaning diets on brain fatty acid composition and FABP7 expression in developing rat brains. The study was carried out at two developmental stages: three weeks postnatal, which is developmentally equivalent to that of a 2–3-year old human, and six weeks postnatal, which is developmentally equivalent to that of a 12–18-year old human. Our data suggest that high levels of DHA in the maternal diet during lactation increases its levels in the infant brain and seems to have a protective effect since levels of DHA in brain can potentially be maintained up to adolescence even when the offspring is weaned to and maintained on a diet that is deficient in DHA. Furthermore, our data suggest that in cases where DHA is not supplied during lactation, it may still be possible to increase its levels in the brain by direct feeding perhaps until adolescence. Finally, there may be an association between brain DHA levels and FABP7 expression, with levels of FABP7 potentially reflecting brain maturation. The relevance of this observation to human health remains to be explored.

## Supplementary Materials

Supplementary materials can be accessed at: http://www.mdpi.com/2072-6643/7/10/5433/s1.
