# Supplementary material for: Long-Term Effect of Docosahexaenoic Acid Feeding on Lipid Composition and Brain Fatty Acid-Binding Protein Expression in Rats
Source: Nutrients. 2015 Oct 22;7(10):8802–17. doi: 10.3390/nu7105433 (PMC4632453; doi:10.3390/nu7105433)
Supplement: Supplementary file 1 [file nutrients-07-05433-s001.docx]

**Supplemental Materials**

|  |
| --- |
|  |

**Figure S1.** Effect of Age on Brain Fatty Acid Composition in Three-week and Six-week Old Pups Fed Cnt diet. (**A**) ω-6 PUFA, ω-3 PUFA and ω-6 PUFA:ω-3 PUFA ratio; and (**B**) arachidonic acid (AA), docosahexaenoic acid (DHA) content and the ratio of AA:DHA in the brains of three and six-week old pups. Pups born to dams which were fed control (Cnt) diet during lactation were fed Cnt diet for three weeks after weaning. Pups were sacrificed at three and six weeks and brain fatty acid composition was analysed. * indicates significant difference from the three-week old pup brains.

© 2015 by the authors; licensee MDPI, Basel, Switzerland. This article is an open access article distributed under the terms and conditions of the Creative Commons by Attribution (CC-BY) license (http://creativecommons.org/licenses/by/4.0/).
